# An Automated System for Physician Trainee Procedure Logging via Electronic Health Records

**DOI:** 10.1001/jamanetworkopen.2023.52370

**Published:** 2024-01-24

**Authors:** Brian Kwan, Jeffery Engel, Brian Steele, Leslie Oyama, Christopher A. Longhurst, Robert El–Kareh, Michelle Daniel, Charles Goldberg, Brian Clay

**Affiliations:** 1 Department of Emergency Medicine, University of California, San Diego, School of Medicine, San Diego; 2 Department of Biomedical Informatics, University of California, San Diego Health, San Diego; 3 Department of Information Services, University of California, San Diego Health, San Diego; 4 Office of Graduate Medical Education, University of California, San Diego Health, San Diego; 5 Office of the Chief Medical Officer and Chief Digital Officer, University of California, San Diego Health, San Diego; 6 Department of Pediatrics, University of California, San Diego, School of Medicine, San Diego; 7 Division of Hospital Medicine, Department of Medicine, University of California, San Diego School of Medicine, San Diego; 8 Office of the Associate Chief Medical Officer for Transformation and Learning, University of California, San Diego Health, San Diego; 9 Office of the Vice Dean for Medical Education, University of California, San Diego, School of Medicine, San Diego; 10 Office of the Associate Dean for Graduate Medical Education, University of California, San Diego, School of Medicine, San Diego; 11 Office of the Associate Chief Medical Officer, University of California, San Diego Health, San Diego

## Abstract

**Importance:**

Procedural proficiency is a core competency for graduate medical education; however, procedural reporting often relies on manual workflows that are duplicative and generate data whose validity and accuracy are difficult to assess. Failure to accurately gather these data can impede learner progression, delay procedures, and negatively impact patient safety.

**Objective:**

To examine accuracy and procedure logging completeness of a system that extracts procedural data from an electronic health record system and uploads these data securely to an application used by many residency programs for accreditation.

**Design, Setting, and Participants:**

This quality improvement study of all emergency medicine resident physicians at University of California, San Diego Health was performed from May 23, 2023, to June 25, 2023.

**Exposures:**

Automated system for procedure data extraction and upload to a residency management software application.

**Main Outcomes and Measures:**

The number of procedures captured by the automated system when running silently compared with manually logged procedures in the same timeframe, as well as accuracy of the data upload.

**Results:**

Forty-seven residents participated in the initial silent assessment of the extraction component of the system. During a 1-year period (May 23, 2022, to May 7, 2023), 4291 procedures were manually logged by residents, compared with 7617 procedures captured by the automated system during the same period, representing a 78% increase. During assessment of the upload component of the system (May 8, 2023, to June 25, 2023), a total of 1353 procedures and patient encounters were evaluated, with the system operating with a sensitivity of 97.4%, specificity of 100%, and overall accuracy of 99.5%.

**Conclusions and Relevance:**

In this quality improvement study of emergency medicine resident physicians, an automated system demonstrated that reliance on self-reported procedure logging resulted in significant procedural underreporting compared with the use of data obtained at the point of performance. Additionally, this system afforded a degree of reliability and validity heretofore absent from the usual after-the-fact procedure logging workflows while using a novel application programming interface–based approach. To our knowledge, this system constitutes the first generalizable implementation of an automated solution to a problem that has existed in graduate medical education for decades.

## Key Points

**Question:**

Does an automated procedure logging system using electronic health record data increase the accuracy and completeness of trainee procedure logging?

**Findings:**

In this quality improvement study with 47 emergency medicine resident physicians, an automated system extracted procedure data from a widely used electronic health record application and uploaded these data to a residency management software application. Compared with manual workflows, the automated system generated a 78% increase in procedures logged during 1 year (4291 to 7617), with an accuracy of 99.5%.

**Meaning:**

These findings suggest that manual procedure logging by trainees may result in substantial underreporting; an automated system may accomplish this task with significantly greater reliability and accuracy.

## Teaser

This quality improvement study examines the accuracy and completeness of emergency resident trainee procedure logging with a novel system that allows the extraction of bedside procedural completion data from a widely used electronic health record.

## Introduction

The ability to plan for, safely execute, and manage complications of bedside procedures is a critical educational component in many residency training programs. Moreover, the Accreditation Council for Graduate Medical Education (ACGME) has explicitly included procedural proficiency among its 6 Core Competencies for graduate medical education.^1^ The need for procedural competency is also delineated in the ACGME Common Program Requirements for many training programs, including internal medicine,^2^ general surgery,^3^ and family medicine.^4^ The requirements for emergency medicine include minimum numbers of specific procedures that must be completed.^5,6^ Accordingly, residency training programs must track the numbers of bedside procedures completed by their trainees.

The typical workflow for reporting procedural completion relies on residents accessing a third-party residency management software (RMS) application to manually document performance. However, this workflow is fraught with difficulties that limit the accuracy and validity of the data.^7,8,9^ Because trainees appropriately prioritize patient care over administrative tasks, manual logging of procedures is frequently done after the fact rather than at the time the procedure was performed. Residents therefore must rely on their memory of the procedure details, which limits the accuracy of the data. Alternatively, they can attempt to record procedure completion on paper or electronically in a manner that satisfies confidentiality concerns, which raises its own set of potential limitations. In addition to the concern regarding the accuracy of manually logged data, trainees often perform procedure logging outside their clinical work time, which has a deleterious impact on wellness. Finally, because it is impractical for trainees to record procedures after every shift given the redundancy inherent in manual logging, recording is often delayed until the end of a clinical rotation, or even until the end of the academic year. For residency program leadership, this makes it difficult to ensure that residents have procedural experience that is commensurate with their stage in training. Moreover, inaccuracies and delays in recording procedures make it difficult for programs to proactively monitor and address any experiential gaps so learners may progress in their training. Inaccurate reporting can also lead to delays in care, as hospital systems often require that nursing staff use procedural completion data to ensure that trainees are “signed off” on a procedure before letting them proceed. Thus, failure to capture the true number of procedures performed by trainees can lead nurses to prevent them from performing the procedure, even if they have met the minimum required threshold for number of procedures performed.

Furthermore, for reasons of continuity of care, medicolegal and regulatory compliance, and coding and billing requirements, residents must document their performance of procedures in the electronic health record (EHR) at the time of completion in the form of a procedure note. Given this requirement, any workflows that rely on separate manual logging of procedures that are already captured in the EHR result in wasted time and effort. In addition, because the manually logged data reside in RMS databases that are separate from the EHR, there is no way to reliably link procedure data with the patient- and care-related information present in the EHR, representing a lost educational opportunity.

This problem has been noted previously, and several publications have demonstrated disparities between EHR and RMS procedure reporting.^10,11^ Efforts to address analogous problems in pediatrics,^12^ neurology,^13^ radiology,^14^ anesthesia,^15^ and medical student clinical rotations^16^ have been reported, although none incorporated RMS upload functionality. The exception to this is the foundational work performed by Seufert and coworkers^17^ in 2011 in which EHR data were used to populate procedure logs. Of note, this work required the development of specialized software and relied on an EHR system not in common use at that time (in approximately 6% of emergency departments [EDs] in the US) or at present.^18^ It was also studied with a before-and-after implementation approach without blinding, which introduced the potential for bias from a Hawthorne effect.

In this article, we describe a novel system that we successfully implemented at our institution to allow emergency medicine resident trainees to have their bedside procedural completion data extracted directly from a widely used EHR (Epic Hyperspace, Epic Systems Corp) using uncomplicated and preexisting EHR functionality. This system is in use at nearly 60% of the teaching hospitals in the US (Epic Systems Corp, written communication, June 5, 2023).^19^ Our system also included functionality to upload these data securely to an RMS application in common use by approximately 10 500 training programs worldwide via its application programming interface (API) (MedHub, written communication, June 7, 2023).

## Methods

This quality improvement study took place at University of California, San Diego Health, the only comprehensive academic health system in San Diego, California, which is affiliated with the University of California, San Diego School of Medicine. Due to the breadth of bedside procedures performed as part of their training, residents in the emergency medicine residency program (a 4-year program with 47 residents) were selected as the target population. The automated system captured bedside procedures performed in the EDs at 2 acute care hospitals, University of California, San Diego Medical Center Hillcrest (a level I trauma center) and Jacobs Medical Center at University of California, San Diego Health (a quaternary care facility), with a total of approximately 90 000 annual visits. Initial data on the capture and extraction component of the system were collected from May 23, 2022, to May 7, 2023.

The EHR build and implementation leveraged existing functionality in Epic Hyperspace. One of the authors (B.K.) executed the EHR build, composed the relational database queries, performed the data analysis, and built the procedure dashboards; another author (J.E.) adapted output from the relational database queries and built the infrastructure for upload via API (eMethods 1 in Supplement 1); configuration changes in the RMS were completed by a separate author (B.S.). The University of California, San Diego Aligning and Coordinating Quality Improvement, Research, and Evaluation Committee^20^ evaluated this quality improvement project and determined that it neither involved human participant research nor required institutional review board review or approval; therefore, patient informed consent was not obtained. The revised Standards for Quality Improvement Reporting Excellence (SQUIRE) reporting guidelines were followed during this investigation (eMethods 2 in Supplement 1).^21^

### System Design

An overview of the strategy used is provided in eFigure 1 in Supplement 1. We began by determining our procedures of interest. If those procedures had associated procedure orders in the EHR, then their associated data were already stored in a well-structured format in the EHR relational database. Procedures that lacked associated procedure orders were addressed by using other standard components of EHR configuration available in Epic Hyperspace. These procedures were either captured by way of a narrator functionality, which facilitates nurse-driven documentation of personnel present and individual events that transpire during an emergent clinical encounter (such as a trauma resuscitation), or by creating structured documentation elements (ie, SmartLists and SmartData Elements) that were added to standard resident note templates. Medical resuscitations were counted when the supervising attending physician indicated (via an embedded SmartList with SmartData Elements) in their note attestation that a patient was critically ill.

The system was implemented on May 23, 2022. Residents were unaware of the presence of the system so that a contemporaneous comparison could be made between the standard manual logging workflow and results obtained from the automated data capture. For procedures to be extracted by the automated system, all 3 of the following conditions had to be satisfied: residents had to create procedure documentation in the EHR, residents had to sign the note containing the procedure documentation, and the note with the procedure documentation had to be attested by the supervising attending physician. Residents manually logged procedures using RMS maintained by a third-party vendor (MedHub). Manually logged procedures were only included in the data analysis if they were performed at 1 of the 2 UCSD EDs, were marked as performed (not supervised, observed, or simulated), and were verified by the supervising attending physician.

Data for procedures captured by the automated system were obtained from queries composed in Structured Query Language and executed against the relational database system within the Epic EHR (Clarity). Procedure extraction was executed weekly after a 1-week delay to allow sufficient time for procedure documentation to be completed. Dashboards were assembled by first preparing the data using Tableau Prep Builder and then building the necessary views in Tableau Desktop (Tableau Software LLC).

Accuracy during the upload phase was determined by individual resident interviews. During these interviews, residents were asked in 1-week intervals to review all procedures uploaded to the RMS system on their behalf and to report if any procedures were missing or incorrectly uploaded. A procedure was counted as a true positive if the automated system captured and uploaded the procedure and the resident verified that the procedure was completed; a procedure was deemed a false negative if the system did not capture or upload the procedure but a resident stated that the procedure was in fact performed; false positives were procedures that the system captured and uploaded but the resident stated were not performed; finally, each emergency department encounter during which no procedures were performed for a patient, and the absence of procedures was verified by the resident, were counted as true negatives. Recall (sensitivity), specificity, and accuracy were calculated in the usual manner.

## Results

**Figure 1.**
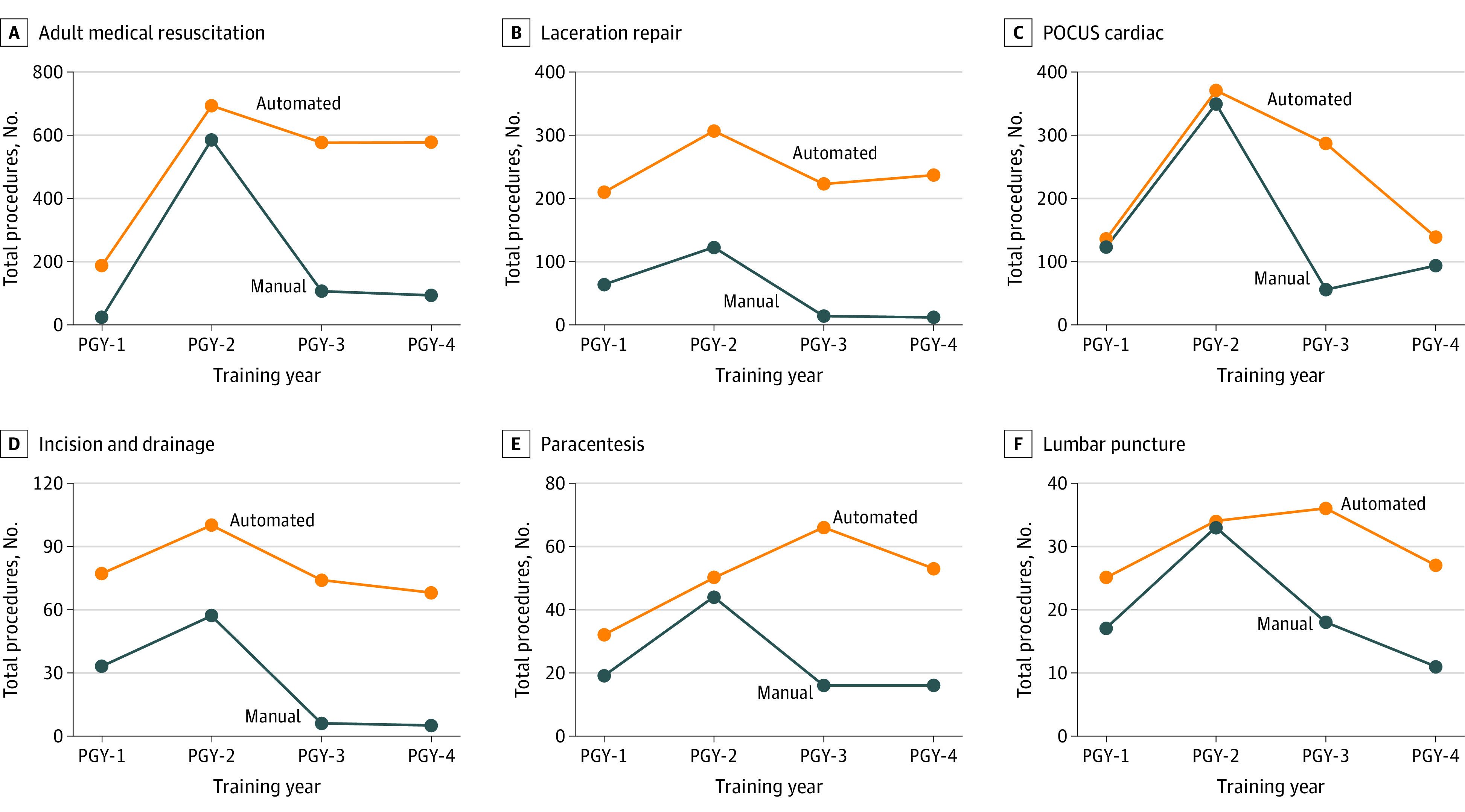
Comparison of Automated and Manual Capture of Procedure Counts for Selected Procedures by Training Year PGY indicates postgraduate year; POCUS, point-of-care ultrasonography.

During the study period, 4291 procedures were manually logged by residents, compared with 7617 procedures captured by the automated system (an increase of 78%). The automated system outperformed the standard workflow involving manual logging for virtually all procedure categories. A comparison of procedures captured with both modalities for the group as a whole and by training year is given in the Table. Figure 1 compares procedure counts by training year from automated and manual capture modalities for a selection of procedures.

**Table.** Comparison of Procedures Captured via Manual vs Automated Workflows by Trainee Year, May 22, 2022, to May 7, 2023

Procedure	No. (%) of procedures by year of training								Total No. of procedures	
	PGY-4 (n = 20/18)^a^		PGY-3 (n = 21/22)^a^		PGY-2 (n = 21/22)^a^		PGY-1 (n = 12/23)^a^			
	Manual	Automated	Manual	Automated	Manual	Automated	Manual	Automated	Manual	Automated
Adult medical resuscitation	94 (11.6)	578 (28.4)	107 (13.2)	577 (28.3)	587 (72.4)	694 (34.1)	23 (2.8)	187 (9.2)	811	2036
Adult trauma resuscitation^b^	72 (24.9)	120 (24.8)	78 (27.0)	298 (61.6)	94 (32.5)	0	45 (15.6)	66 (13.6)	289	484
Intubation	33 (10.7)	27 (8.6)	31 (10.1)	34 (10.9)	237 (76.9)	241 (77.0)	7 (2.3)	11 (3.5)	308	313
Procedural sedation	37 (32.5)	51 (37.8)	28 (24.6)	38 (28.1)	41 (36.0)	35 (25.9)	8 (7.0)	11 (8.1)	114	135
Arterial line	13 (17)	24 (26)	9 (12)	17 (19)	46 (60)	35 (38)	8 (11)	16 (17)	76	92
Central line	33 (20.1)	49 (23.9)	20 (12.2)	35 (17.1)	81 (49.4)	81 (39.5)	30 (18.3)	40 (19.5)	164	205
Intraosseous line placement	0 (0)	0 (0)	2 (11)	0 (0)	11 (61)	4 (50)	5 (28)	4 (50)	18	8
Transvenous pacing	9 (41)	3 (37.5)	5 (23)	2 (25)	8 (36)	3 (37.5)	0 (0)	0 (0)	22	8
Cardioversion	15 (38)	26 (52)	10 (26)	15 (30)	13 (33)	6 (12)	1 (3)	3 (6)	39	50
Arthrocentesis	10 (24)	22 (31)	4 (9)	16 (23)	17 (41)	16 (23)	11 (26)	16 (23)	42	70
Fracture or dislocation reduction	29 (26.1)	44 (32.4)	22 (19.8)	43 (31.6)	47 (42.3)	25 (18.4)	13 (11.8)	24 (17.6)	111	136
Laceration repair	12 (5.6)	237 (24.3)	14 (6.6)	223 (22.8)	123 (57.8)	307 (31.4)	64 (30.0)	210 (21.5)	213	977
Nerve block	4 (6)	19 (20)	12 (18)	29 (31)	38 (59)	30 (32)	11 (17)	16 (17)	65	94
Incision and drainage	5 (5.0)	68 (21.3)	6 (5.9)	74 (23.2)	57 (56.4)	100 (31.4)	33 (32.7)	77 (24.1)	101	319
Chest tube	14 (37)	13 (38)	5 (13)	3 (9)	16 (42)	16 (47)	3 (8)	2 (6)	38	34
Paracentesis	16 (17)	53 (26.4)	16 (17)	66 (32.8)	44 (46)	50 (24.9)	19 (20)	32 (15.9)	95	201
Thoracentesis	3 (23)	8 (33)	4 (31)	9 (38)	4 (31)	6 (25)	2 (15)	1 (4)	13	24
Lumbar puncture	11 (14)	27 (22.1)	18 (23)	36 (29.5)	33 (42)	34 (27.9)	17 (21)	25 (20.5)	79	122
POCUS cardiac	94 (15.1)	139 (14.8)	56 (9.0)	287 (30.8)	350 (56.2)	371 (39.8)	123 (19.7)	136 (14.6)	623	933
POCUS lung	14 (26)	28 (15.2)	4 (7)	49 (26.6)	31 (56)	75 (40.8)	6 (11)	32 (17.4)	55	184
POCUS soft tissue, DVT, musculoskeletal, or other	73 (28.7)	43 (13.7)	24 (9.4)	82 (26.0)	106 (41.7)	129 (41.0)	51 (20.1)	61 (19.4)	254	315
POCUS abdomen	238 (32.9)	157 (20.4)	53 (7.3)	197 (25.6)	294 (40.6)	267 (34.7)	139 (19.2)	148 (19.3)	724	769
POCUS ocular	1 (10)	17 (21)	3 (30)	34 (42)	6 (60)	22 (27)	0 (0)	8 (10)	10	81
Ultrasonography-guided intravenous catheter placement	4 (15)	7 (26)	0 (0)	6 (22)	16 (59)	8 (30)	7 (26)	6 (22)	27	27

Abbreviations: DVT, deep venous thrombosis; POCUS, point-of-care ultrasonography.

^a^ Reported as the number of residents who manually logged at least 1 procedure/had at least 1 procedure extracted via the automated system. The number of residents may be greater than the size of a single class since the study period included portions of 2 academic years.

^b^ On the basis of institutional deployment of the trauma narrator functionality beginning on October 4, 2022.

**Figure 2.**
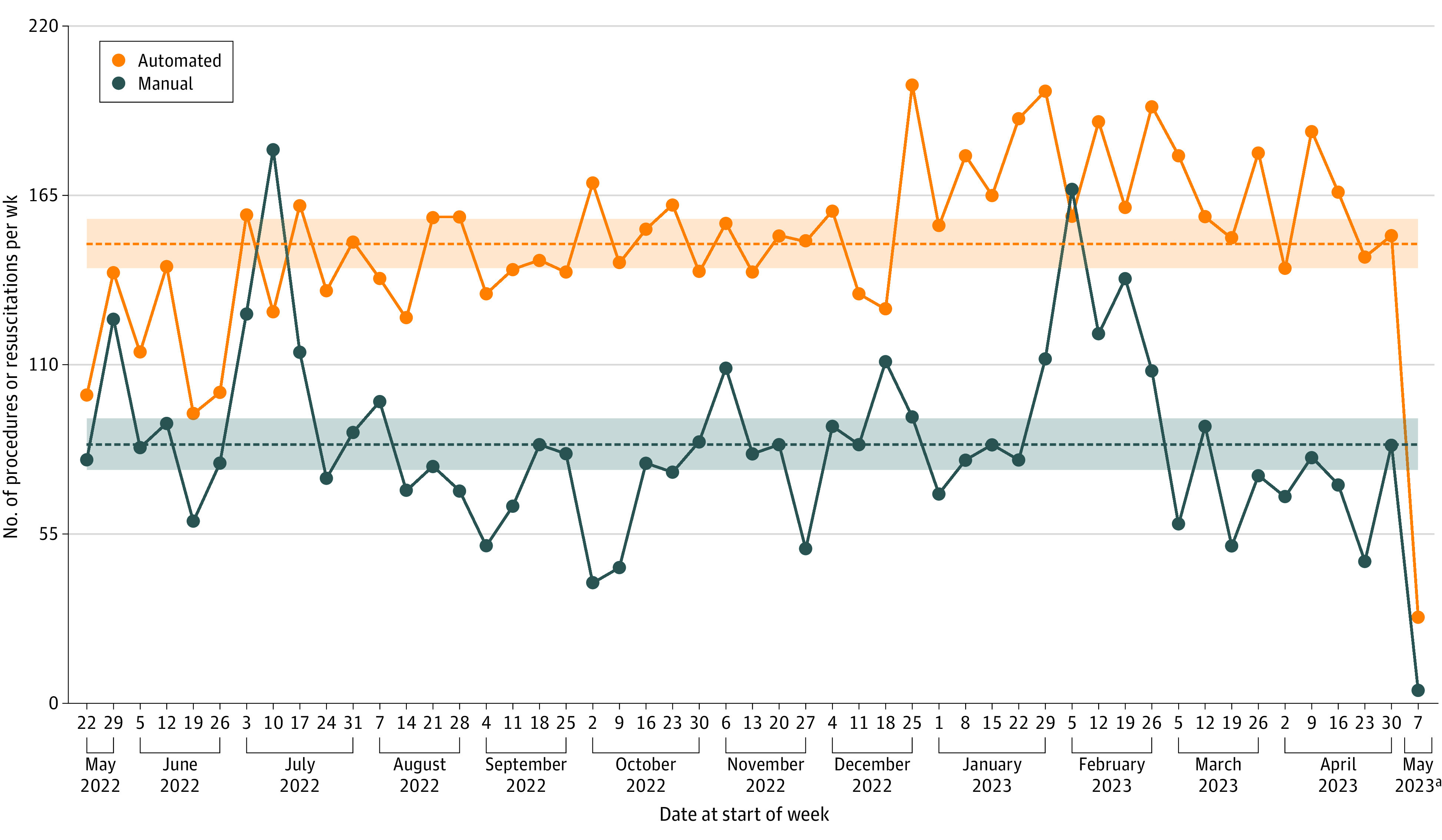
Procedure and Resuscitation Counts by Week, May 22, 2022, to May 7, 2023 For each modality, dotted lines represent the mean number of procedures logged per week, and the shaded area represents the 95% CIs. ^a^For the week of May 7, 2023, only 1 day of procedures were logged.

We plotted the number of procedures completed per week in the same timeframe, with the results shown in Figure 2. Manual logging resulted in a mean (SD) of 84 (30) procedures per week (95% CI, 76-93), whereas the logging system captured a mean (SD) of 149 (29) procedures per week (95% CI, 141-157). Once we established that the extraction and capture portion of the system was functioning successfully, we turned our attention to development of the API-based RMS upload component of the system, which was deployed on May 8, 2023. Through June 25, 2023, there were 266 true-positive results, 7 false-negative results, 0 false-positive results, and 1080 true-negative results. From these values, we calculated a recall (sensitivity) of 97.4%, specificity of 100%, and an overall accuracy of 99.5%. As part of a plan to distribute procedural performance data to residency program leadership as well as to individual trainees, we built dashboards to show the pertinent information. A sample of the procedure dashboards can be found in eFigure 2 in Supplement 1).

## Discussion

The existence of discrepancies between manual procedure logging and actual numbers of procedures performed by trainees represents a longstanding issue in graduate medical education. We believe our system has demonstrated the ability to address this issue in a manner readily adaptable to a wide variety of other training programs and organizations.

An interesting phenomenon illustrated in Figure 1 is that with multiple procedures there was a notable decrease in manual logging on transitioning from junior to senior resident status (from postgraduate year 2 to postgraduate year 3), whereas the automated system demonstrated a much less significant decrease, or even an increase. One possible explanation for this is that with manual workflows, incentives to continue logging are diminished once a learner believes that they have met the minimum required number of procedures. Consequently, a substantial amount of procedural experience is lost. With automated logging, this issue is mitigated, because the data that are collected more closely reflect the clinical reality of what learners are actually doing during patient care.

Of note, in several categories of procedures, the numbers of procedures manually logged exceeded those captured by the automated system, including intraosseous catheter placement, transvenous pacing, and chest tube placement. Several residents were interviewed about these discrepancies and indicated that for 2 of these procedures (intraosseous catheter placement and transvenous pacing), they were not aware that documentation options existed in the EHR for these procedures; as with many EHR features, end-user education is often a key component that influences the extent of use. For chest tube placement, the difference in performance of the automated and manual systems was negligible (34 vs 38 procedures, respectively). In an individual interview, one resident indicated that while entering many procedures in one sitting, they had inadvertently manually logged a chest tube placement as being performed at one of the locations where the system was in operation when, in fact, the procedure was performed during an off-site rotation. This example highlights several of the shortcomings of relying on manually logged data. First, it is relatively easy to make an error when manually entering data. Second, there is often no way to assess the validity of the manually logged data outside of discussions of individual procedures with individual residents, which is clearly not practical to do at scale. In addition, this type of error would be impossible with the automated system in place, given that offsite procedures would not be captured. Third, manual entry processes incentivize entry of large numbers of procedures at once, limiting the ability to assess procedural completion at frequent intervals. For example, there are several maxima in manually logged procedures noted in Figure 2. The explanation for these anomalies is unclear, but it is notable that the first (week of May 29, 2022) coincided with the start of the last clinical block of the academic year, and the second (week of July 10, 2022) occurred shortly after the start of the new academic year. This phenomenon results in much larger temporal variability in manual logging, as demonstrated in Figure 1 and by the fact that the 95% CIs of the mean number of procedures logged per week were roughly equal between modalities, even though the automated system captured nearly 80% more procedures per week.

With respect to the 7 false-negative procedures (those procedures not captured by the automated system but which a resident reported as performed), 6 of these were procedures in which the resident had completed documentation in the EHR, but those notes had not yet been attested by the supervising attending physician. In the remaining instance, the resident forgot to complete the procedure documentation in the EHR.

Regardless of which method (narrator or structured documentation elements [ie, SmartLists or SmartData Elements]) was chosen to capture procedural data (eFigure 1 in Supplement 1), there was little or no need to make changes to the preexisting EHR documentation workflow, which was attractive from a quality improvement perspective. SmartLists are well suited for procedure documentation and can be built rapidly. This feature makes it possible to easily capture both extant procedures lacking documentation templates and new procedures with only minor edits to standard procedure note templates.

There are several areas in which having accurate and valid procedure completion data fills an educational void, particularly with respect to the nascent field of precision medical education.^22^ First, by efficiently and accurately collecting data on resident procedure completion and disseminating it to learners and residency program leadership, this system holds great potential to customize coaching for individual learners. Second, in place of the usual ex post facto end-rotation or end–academic year updates, this system enables frequent population-level guidance to residency program leadership on the extent of procedural experience without creating an imposition on trainees to reproduce their EHR documentation by manually logging procedures in real time.^23^ Finally, recent literature has investigated the extent to which bias plays a role in evaluation and procedural completion in certain settings.^24,25^ By making it possible to collect reliable and accurate data on procedures that can be reported simultaneously with the vast quantities of other data stored in the EHR, this system would enable further investigations as to gender, racial, ethnic, or other disparities that may exist with regard to procedural performance among residents.

### Limitations

There are several limitations to this work. First, the study was implemented at a single academic medical center. Second, the test population consisted of residents in a single training program. Third, the procedures captured were limited to bedside procedures (ie, those not performed in an operating room). Fourth, a single EHR and RMS application were used to deploy the system.

## Conclusions

This novel system for automated resident procedure logging based on data stored in an EHR allows for frequent and accurate assessment of progress for procedural completion requirements, something not possible with manual logging in which large numbers of procedures are often entered in short bursts, often well after the time of completion. Furthermore, because data were obtained directly from EHR-associated relational databases, our system enables straightforward and simultaneous reporting on any other desired information stored in the EHR along with procedure data. Emphases on competency-based medical education from the ACGME, as well as the more recent focus on precision medical education by organizations such as the American Medical Association, will require valid, relevant, and accessible data, which we believe this system uniquely enables in a generalizable manner for resident-performed procedures.

## Notes

Supplement 1. **eMethods 1.** Specifications for Procedure Data Transmission to MedHub Residency Management Software **eMethods 2.** SQUIRE Guidelines Reporting Checklist **eFigure 1.** Schematic Depiction of Strategy for Implementation of Automated Procedure Logging System **eFigure 2.** Sample of Dashboard Built to Display Procedure Completion Data Obtained From Automated System

Supplement 2.
Data Sharing Statement
