# Supplementary material for: An Automated System for Physician Trainee Procedure Logging via Electronic Health Records
Source: JAMA Netw Open. 2024 Jan 24;7(1):e2352370. doi: 10.1001/jamanetworkopen.2023.52370 (PMC10809018; doi:10.1001/jamanetworkopen.2023.52370)
Supplement: Supplement 1. — eMethods 1. Specifications for Procedure Data Transmission to MedHub Residency Management Software eMethods 2. SQUIRE Guidelines Reporting Checklist eFigure 1. Schematic Depiction of Strategy for Implementation of Automated Procedure Logging System eFigure 2. Sample of Dashboard Built to Display Procedure Completion Data Obtained From Automated System [file jamanetwopen-e2352370-s001.pdf]

Supplementary Online Content

Kwan B, Engel J, Steele B, et al. An automated system for physician trainee procedure logging via electronic health records. *JAMA Netw Open*. 2024;7(1):e2352370. doi:10.1001/jamanetworkopen.2023.52370

eMethods 1. Specifications for Procedure Data Transmission to MedHub Residency Management Software

eMethods 2. SQUIRE Guidelines Reporting Checklist

eFigure 1. Schematic Depiction of Strategy for Implementation of Automated Procedure Loging System

eFigure 2. Sample of Dashboard Built to Display Procedure Completion Data Obtained From Automated System

This supplementary material has been provided by the authors to give readers additional information about their work.

© 2024 Kwan B et al. *JAMA Network Open.*

eSupplement 1. Specifications for Procedure Data Transmission to MedHub Residency Management Software

This document contains a description of the system we built to send procedure completion data to the MedHub (MH) Residency Management Software (RMS) database via its web service API. It is based on a program written in C# and relies on the MH public API specifications at https://api-docs.medhub.com. We worked with a technical representative from MH to identify and understand the needed API calls and data structures, and we performed initial testing and troubleshooting of the API calls in the Postman utility. API calls to MH require a client ID, client key, timestamp, and parameters to be encrypted and sent to the MH endpoint URL.

We set up an intermediate data processing SQL Server database for temporary data storage and processing. From initial testing of queries, we created tables for each of four types of procedures queried from different sources in the EHR:

1. Point–of–care ultrasound (POCUS) studies
2. Medical resuscitations
3. Trauma resuscitations
4. All other procedures

Our process included a delay to give residents enough time to complete their documentation of procedures in the EHR, and for supervising attendings to attest the procedure documentation. At the end of the delay period, we first ran queries to pull each of the above types of procedure data from the EHR data warehouse into the above tables in the data processing database between a specified start and end date.

© 2024 Kwan B et al. *JAMA Network Open.*

Next, we made API web service calls to the MH database to pull in MH data necessary to match National Provider Identifier (NPI) IDs and procedure IDs between the EHR and MH. Although the NPI is the common ID between the systems for both residents and supervisors, MH user IDs must be sent when using the API web service calls. We created a translation table to match from NPI in the EHR to NPI in MH to finally obtain the MH user ID.

To match procedures and avoid the need for a separate translation table, we made use of the cpt_code field in MH that contained no data in our instance of MH. We used the values assigned to each procedure from the PROC_ID column in the CLARITY_EAP table in Clarity if those values existed; otherwise, we used other related numerical values to identify each procedure (*i.e.*, the .1 of the SmartData Element if one was used to identify a specific procedure)

The relevant API calls included:

1) users/residents – takes parameter of program ID – returns a list of all residents in that program, including MH user ID, name, etc., and iterates through the returned list of all residents calling the following to obtain the needed NPI
2) users/residentInfo – takes parameter of userID – returns NPI
3) procedures/supervisors – takes a parameter of program ID and an optional parameter of “offset” – returns MH user ID, name, etc. This MH call returns a maximum of 3000 records per call, so if 3000 are returned, then increment and send parameter “offset” by 3000 until less than 3000 are returned. Iterate through the returned list of all faculty calling the following to obtain NPI

4) users/facultyInfo – takes userID – returns NPI. If no faculty NPI is found using this call, then use the next call, since some faculty NPIs may be found in residentInfo:
5) users/residentInfo – (see above)
6) procedures/procedureTypes – takes programID – returns typeID, procedure_name, cpt_code, etc.

Each API call returns data in JSON format. We used the Newtonsoft.Json library to deserialize the JSON into data classes containing properties for each data field returned. We used generics to create utility methods to process the different classes returned and reflection within those methods to operate on the properties of each data class (for instance, to convert a JSON list of residents returned to a C# DataTable, and then do a SQL bulk copy of the table into the data processing database table).

Queries in the database then match and translate NPIs to obtain MH user IDs, match CPT codes from the EHR with procedure typeID in MH, and return a table of rows with Date, MH resident ID, MH faculty ID, MH proc ID, patient gender, and patient age, to be sent as parameters into MH using the following combination of API calls:

1) procedures/record – returns a log ID – takes the following parameters in the following format:
   {"userID":<MHResidentUserID>,"programID":<MHProgramID>,"locationID":1, "supervisorID":<MHFacultyUserID>,"date":"<MM/DD/YYYY>","mobile":false, "fields":{"patient_gender":"<M, F>","patient_age":"<ageYrs>"}}
2) procedures/appendProcedure – returns a procedure ID – takes the following parameters in the following format:

© 2024 Kwan B et al. *JAMA Network Open.*

{"logID":<log ID returned above>,"typeID":<MHProcedureTypeID>,"role":1}

We also generated reports that show any residents or faculty that failed to find a match between systems and procedures that therefore cannot be sent into MH and risk being missed. This enables review of unmatched procedures for troubleshooting purposes. The most common reason a match was not made was that supervising attendings did not have NPI values entered in MH.

The C# program described was compiled into a Windows service to be run automatically on a schedule.

© 2024 Kwan B et al. *JAMA Network Open.*

eSupplement 2. SQUIRE Guidelines Reporting Checklist

Reporting checklist for quality improvement in health care.

Based on the SQUIRE guidelines.

Instructions to authors

Complete this checklist by entering the page numbers from your manuscript where readers will find each of the items listed below.

Your article may not currently address all the items on the checklist. Please modify your text to include the missing information. If you are certain that an item does not apply, please write "n/a" and provide a short explanation.

Upload your completed checklist as an extra file when you submit to a journal.

In your methods section, say that you used the SQUIREreporting guidelines, and cite them as:

Ogrinc G, Davies L, Goodman D, Batalden P, Davidoff F, Stevens D. SQUIRE 2.0 (Standards for QUality Improvement Reporting Excellence): revised publication guidelines from a detailed consensus process

		Reporting Item	Page Number
**Title**			
	#1	Indicate that the manuscript concerns an initiative to improve healthcare (broadly defined to include the quality, safety, effectiveness, patientcenteredness, timeliness, cost, efficiency, and equity of healthcare)	~~1~~2
**Abstract**			
	#02a	Provide adequate information to aid in searching and indexing	4
	#02b	Summarize all key information from various sections of the text using the abstract format of the intended publication or a structured summary such as: background, local problem, methods, interventions, results, conclusions	4-5
**Introduction**			
Problem description	#3	Nature and significance of the local problem	6-7
Available knowledge	#4	Summary of what is currently known about the problem, including relevant previous studies	7-8
Rationale	#5	Informal or formal frameworks, models, concepts, and / or theories used to explain the problem, any reasons or assumptions that were used to develop the intervention(s), and reasons why the intervention(s) was expected to work	8
Specific aims	#6	Purpose of the project and of this report	4, 8

© 2024 Kwan B et al. *JAMA Network Open.*

**Methods**			
Context	#7	Contextual elements considered important at the outset of introducing the intervention(s)	8-9
Intervention(s)	#08a	Description of the intervention(s) in sufficient detail that others could reproduce it	9-11
Intervention(s)	#08b	Specifics of the team involved in the work	9
Study of the Intervention(s)	#09a	Approach chosen for assessing the impact of the intervention(s)	10-11
Study of the Intervention(s)	#09b	Approach used to establish whether the observed outcomes were due to the intervention(s)	10-11
Measures	#10a	Measures chosen for studying processes and outcomes of the intervention(s), including rationale for choosing them, their operational definitions, and their validity and reliability	10-11
Measures	#10b	Description of the approach to the ongoing assessment of contextual elements that contributed to the success, failure, efficiency, and cost	11
Measures	#10c	Methods employed for assessing completeness and accuracy of data	11
Analysis	#11a	Qualitative and quantitative methods used to draw inferences from the data	10-11
Analysis	#11b	Methods for understanding variation within the data, including the effects of time as a variable	11
Ethical considerations	#12	Ethical aspects of implementing and studying the intervention(s) and how they were addressed, including, but not limited to, formal ethics review and potential conflict(s) of interest	9
**Results**			
	#13a	Initial steps of the intervention(s) and their evolution over time (e.g., time-line diagram, flow chart, or table), including modifications made to the intervention during the project	~~16-17~~12–13
	#13b	Details of the process measures and outcome	~~11~~12–13
	#13c	Contextual elements that interacted with the intervention(s)	12-13
	#13d	Observed associations between outcomes, interventions, and relevant contextual elements	12-13
	#13e	Unintended consequences such as unexpected benefits, problems, failures, or costs associated with the intervention(s).	12-13
	#13f	Details about missing data	12-13
**Discussion**			
Summary	#14a	Key findings, including relevance to the rationale and specific aims	11, 13-14
Summary	#14b	Particular strengths of the project	13-14

© 2024 Kwan B et al. *JAMA Network Open.*

Interpretation	#15a	Nature of the association between the intervention(s) and the outcomes	12-14
Interpretation	#15b	Comparison of results with findings from other publications	7-8, 11
Interpretation	#15c	Impact of the project on people and systems	14
Interpretation	#15d	Reasons for any differences between observed and anticipated outcomes, including the influence of context	12-13
Interpretation	#15e	Costs and strategic trade-offs, including opportunity costs	N/A
Limitations	#16a	Limits to the generalizability of the work	~~14~~16
Limitations	#16b	Factors that might have limited internal validity such as confounding, bias, or imprecision in the design, methods, measurement, or analysis	~~14~~16
Limitations	#16c	Efforts made to minimize and adjust for limitations	N/A
Conclusion	#17a	Usefulness of the work	~~15~~13
Conclusion	#17b	Sustainability	~~15~~13
Conclusion	#17c	Potential for spread to other contexts	~~15~~13
Conclusion	#17d	Implications for practice and for further study in the field	1~~4-15~~6–17
Conclusion	#17e	Suggested next steps	16–1~~4-15~~7
**Other information**			
Funding	#18	Sources of funding that supported this work. Role, if any, of the funding organization in the design, implementation, interpretation, and reporting	N/A

The SQUIRE 2.0 checklist is distributed under the terms of the Creative Commons Attribution License CC BY-NC 4.0. This checklist was completed on 24. August 2023 using https://www.goodreports.org/, a tool made by the EQUATOR Network in collaboration with Penelope.ai

© 2024 Kwan B et al. *JAMA Network Open.*

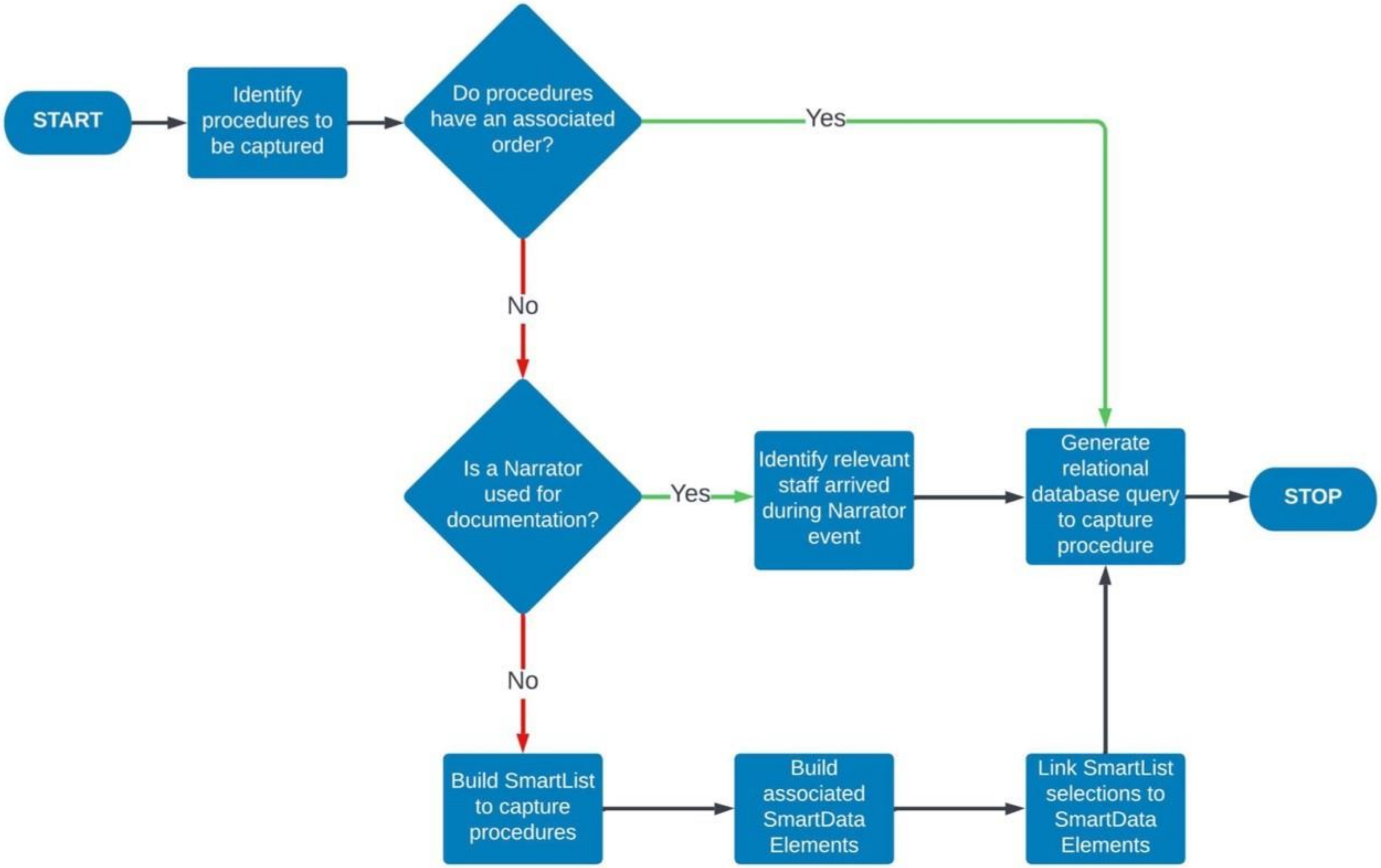

eFigure 1. Design and implementation strategy used for automated resident procedure logging system.

© 2024 Kwan B et al. *JAMA Network Open*.

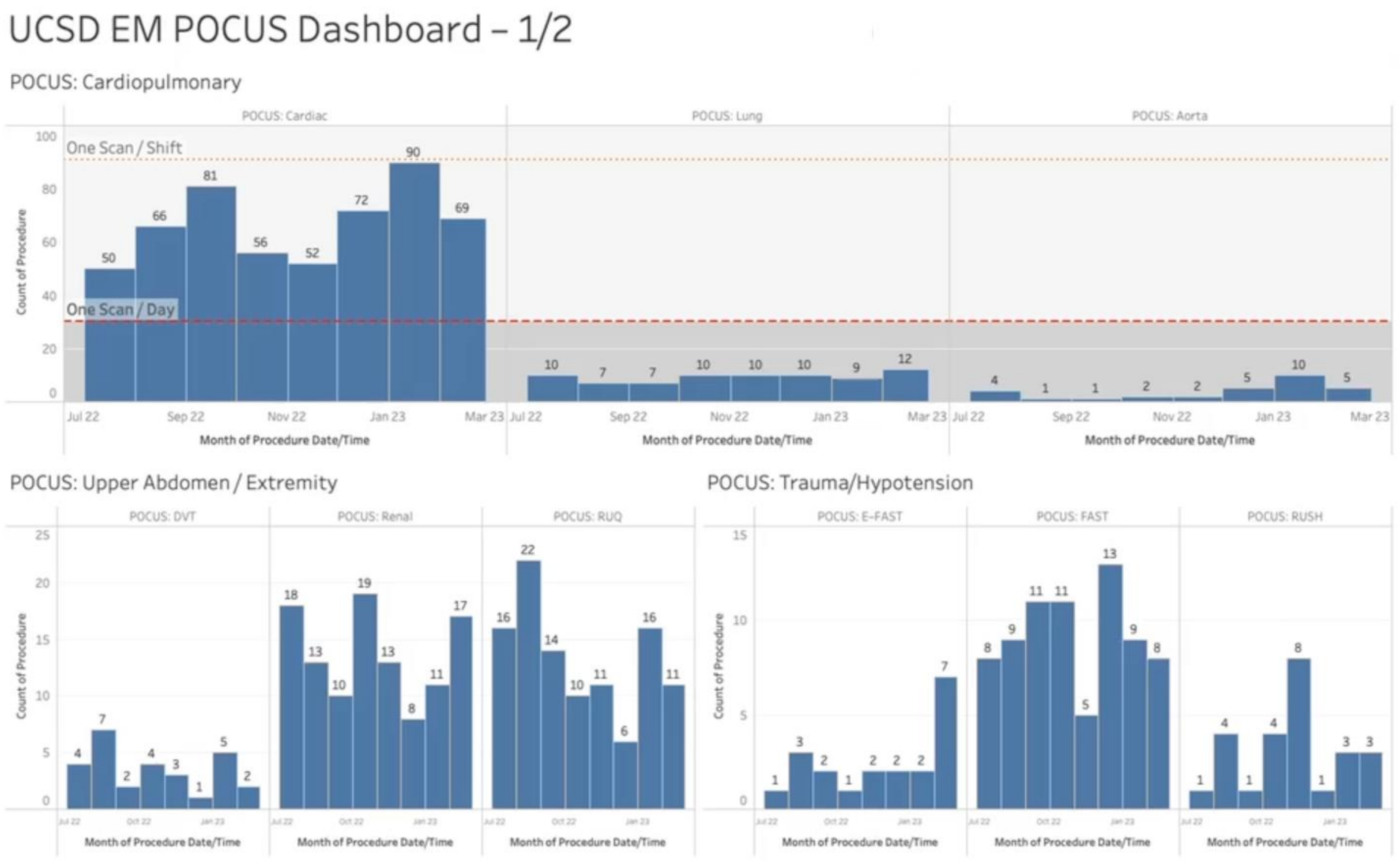

eFigure 2. Sample of a dashboard built as a vehicle to disseminate procedural completion data obtained from the automated system. The x-axis in each subplot represents the month of the procedure performance, and the y-axis

© 2024 Kwan B et al. *JAMA Network Open.*

numbers of procedures. Each subplot represents counts of a different procedure (*e.g.*, POCUS Cardiac, POCUS Lung, POCUS Aorta, *etc.*)

© 2024 Kwan B et al. *JAMA Network Open.*

123

© 2024 Kwan B et al. *JAMA Network Open.*
