# Supplementary material for: An Automated System for Physician Trainee Procedure Logging via Electronic Health Records
Source: JAMA Netw Open. 2024 Jan 24;7(1):e2352370. doi: 10.1001/jamanetworkopen.2023.52370 (PMC10809018; doi:10.1001/jamanetworkopen.2023.52370)
Supplement: Supplement 2. — Data Sharing Statement [file jamanetwopen-e2352370-s002.pdf]

Data Sharing Statement

Kwan. An Automated System for Physician Trainee Procedure Logging via Electronic Health Records. *JAMA Netw Open*. Published January 24, 2024.
doi:10.1001/jamanetworkopen.2023.52370

Data
Data available: No

Additional Information
Explanation for why data not available: To remain in compliance with both HIPAA and FERPA requirements
